# Editorial, special issue on “Advances in Robust Statistics”

**DOI:** 10.1007/s40300-021-00213-w

**Published:** 2021-06-28

**Authors:** Marco Riani, Mia Hubert

**Affiliations:** 1 Dipartimento di Scienze Economiche e Aziendali and Interdepartmental Centre for Robust Statistics, Università di Parma, Via Kennedy 6, 43100 Parma, Italy; 2 Department of Mathematics, Section of Statistics and Data Science, KU Leuven, Celestijnenlaan 200B, 3001 Leuven, Belgium

**Keywords:** Outliers, Influence function, Breakdown point

## Abstract

Starting with 2020 volume, the journal Metron has decided to celebrate the centenary since its foundation with three special issues. This volume is dedicated to robust statistics. A striking feature of most applied statistical analyses is the use of methods that are well known to be sensitive to outliers or to other departures from the postulated model. Robust statistical methods provide useful tools for reducing this sensitivity, through the detection of the outliers by first fitting the majority of the data and then by flagging deviant data points. The six papers in this issue cover a wide orientation in all fields of robustness. This editorial first provides some facts about the history and current state of robust statistics and then summarizes the contents of each paper.

## Introduction

This volume of Metron is one of the three special issues celebrating the hundredth anniversary of the journal. We are grateful to the Editor-in-Chief Professor Marco Alfò for recognizing the importance of robustness and for giving us the possibility to bring it under the attention of all Metron readers. With this volume we hope to increase the interest in robust methodology even more.

Since real data frequently depart from the assumptions behind the models that are used to derive tests and other statistical procedures, it is of utmost importance to dispose of robust procedures that behave well under, usually small, departures from these assumptions. Robust statistics also aims at detecting outlying observations by searching for the model fitted by the majority of the data.

Whereas an early use of the term robustness is due to Box [4], the current understanding of robust statistics is much more the creation of Tukey, starting with Tukey [18], and of Huber [7]. To quantify the degree of robustness, the influence function and the breakdown point are introduced as key mathematical tools, see Hampel et al. [5], Portnoy and He [13] and Stigler [16] provide nice reviews.

The earliest book-length reference is Andrews et al. [1] (the Princeton Robustness Study). Now, a further 50 years on, there are at least six books about robust statistics with over 1000 citations in Google Scholar. At the time of writing, the most highly cited is Huber [8] (and its second edition Huber and Ronchetti [9]). The others, in citation order, are Rousseeuw and Leroy [14], Hampel et al. [5], Maronna et al. [10] (and its second edition) and Hoaglin et al. [6].

As mentioned by Elvezio Ronchetti in his paper in this volume, about 8000 journal papers on robustness have appeared in the period 1978–2017, and now yearly about 200 new papers are published. Over the years also Metron has regularly published work about robustness, such as Borroni and Cifarelli [3], Tóth and Somorčík [17] and Sinha [15] to name a few.

Applications of robust methods are numerous. Many researchers in a wide variety of research domains include robust procedures as part of their statistical analysis. Also the public and the private sector recognize more and more the benefit of using methods that detect anomalies in the data. We can mention two contexts in the public sector. During the COVID-19 pandemic the European Commission (EC) had to monitor constantly the supply of critical commodities in the European Union. Thanks to the availability of robust methods for cluster-wise regression and time series analysis the EC could deploy in record time (less than 1 month, between May and June 2020) a system for monitoring the supply chain of critical commodities, such as face masks, respirators, medicines and, more recently, vaccines (Perrotta et al. [11]). In particular, the robust analysis of the EU trade of face masks supported in an unequivocal way the need for introducing new codes for the import-export of protective face masks, which were introduced in the legislation in October 2020 after careful examination by the competent regulation authorities. A second example relates to the anti-fraud policy of the European Union. Also in this context robust methods are extensively applied (Perrotta et al. [12]) and the potential impact on the protection of the EU budget is enormous, as the special report of the European Court of Auditor claims in relation to an undervaluation fraud case worth billions of euros detected using a form of “Outlier-Free Average Prices”^1^. Anomaly detection techniques based on robust statistics are also applied by Baesens et al. [2] to detect fraud in a real payment transactions data set from a large European bank.

The success of robust methods goes hand in hand with the availability of reliable software. Many researchers have contributed significantly to provide open-source software with the goal to disseminate the various methods of robust statistics. The R software contains more than 200 packages mentioning ‘robust’ in their description, such as the packages *robustbase, rrcov, robustHD, RobStatTM, robfilter, cellWise, roahd, robCompositions, mrfDepth, tclust*, all available at https://cran.r-project.org.

MATLAB code is available via *FSDA Flexible Statistics and Data Analysis* (https://www.mathworks.com/matlabcentral/fileexchange/72999-fsda) and

*LIBRA* (https://wis.kuleuven.be/statdatascience/robust/LIBRA/).

Initially, most contributions in robustness were related to the most fundamental statistical models, such as location, scale, covariance, linear regression and time series. Later, partly thanks to the development of fast algorithms and higher computing power, research moved on towards more advanced multivariate methods (among which classification and clustering) and higher level regression settings such as generalized linear models. In the past two decades more and more challenging problems were tackled, such as high-dimensional and big data. Despite all this activity, much remains to be done to cope with modern complex problems, like for example those occurring in circular data, functional data and count time series. This special issue goes in the direction of trying to fill such gaps, containing six papers dealing with quite distinct topics.

## Description of the papers

*Elvezio Ronchetti* contributes the first paper of this METRON special issue with a review entitled “The main contributions of robust statistics to statistical science and a new challenge”. He traces the development of robust statistics through its main contributions which have penetrated mainstream statistics and focuses on basic concepts that have become standard ideas and tools in modern statistics. In this paper there is both a historical overview, embedded in the modern literature, together with indications of future challenges in this field. He encourages us to include aspects of robust statistics in undergraduate and graduate courses in statistics. As one of the referees wrote, this is a “well written, perfect language, rigorous in the notation, short and concise review paper”.

*Hanan Elsaied and Roland Fried* contribute with a paper entitled “On robust estimation of negative binomial INARCH models”. The authors propose robust estimators for the parameters of negative binomial INARCH models. These are useful time series models for integer data. More specifically, these authors address from a robust point of view the estimation of INARCH models for count time series, where each observation, conditionally on its past, follows a negative binomial distribution with a constant scale parameter, and the conditional mean depends linearly on previous observations. The authors develop several robust estimators, some of them being computationally fast modifications of methods of moments while others are efficient modifications of conditional maximum likelihood. The usefulness of the proposed methods is illustrated by a real data example and a series of simulation studies.

*Graciela Boente and Matías Salibián-Barrera* study the problem of “Robust functional principal components for sparse longitudinal data”. In this paper the authors initially review existing methods for robust functional principal component analysis (FPCA). Then they propose a new method for FPCA that can be applied to longitudinal data where only a few observations per trajectory are available. This method is robust against the presence of atypical observations, and can also be used to derive a new non-robust FPCA approach for sparsely observed functional data. The finite sample performance of the proposal is explored through a simulation study which not only shows that the robust method outperforms existing alternatives when the data are contaminated, but also highlights that for samples that do not contain outliers the non-robust variant of their proposal compares favourably to the existing alternative in the literature.

*Thomas Ortner, Peter Filzmoser, Maia Rohm, Sarka Brodinova and Christian Breiteneder* present a new approach called “Local projections for high-dimensional outlier detection”. The core aim of their proposal is to measure the outlyingness of observations avoiding any assumptions on the underlying data distribution and being able to cope with high-dimensional datasets with fewer observations than variables (flat data structures). To achieve this purpose the authors combine the concepts of the Local Outlier Factor (LOF) originating from the field of computer science, and ROBPCA, a robust principal component analysis-based approach for outlier detection coming from the field of robust statistics. The final result is an algorithm that is robust towards noise variables and is also capable of performing outlier detection in multi-group situations. Experiments with simulated and real data demonstrate the usefulness of their method when compared to existing outlier detection algorithms.

*Himanshu Rai, Sanjeev K. Tomer and Anoop Chaturvedi* contribute with a paper entitled “Robust estimation with variational Bayes in presence of competing risks”. Variational Bayes is a method from machine learning which can provide a good approximation to the intractable posterior density function. It converges fast and works efficiently for large data sets. In this paper, the authors use this approach for robust Bayesian estimation of cause-specific quantities using competing risk data with missing causes. They consider the contamination class of prior distributions for the parameter of interest and discuss the implementation of the method in order to select a prior in a data-dependent fashion leading to a robust posterior distribution. Using real and simulated data, the authors provide evidence that the suggested approach provides robust Bayes estimates of the parameters of interest, namely cause-specific hazard and the cumulative incidence function.

*Giovanni Saraceno, Claudio Agostinelli and Luca Greco* consider circular data in “Robust Estimation for Multivariate Wrapped Models”. This paper deals with the analysis of multivariate circular observations where the quantity of interest is measured as a direction or when an instrument such as a compass is used. Circular (or directional) data can be seen as points on the unit circle and represented by angles. These data can be successfully modeled by using appropriate Wrapped distributions on the unit circle. In this paper the authors use a weighted likelihood technique for robust estimation of a multivariate Wrapped distribution of data points scattered on a *p*-dimensional torus. A set of data dependent weights based on the Pearson residuals are built and the corresponding weighted likelihood estimating equations are solved. In particular, robust estimation is carried out by using a Classification EM algorithm whose M-step is enhanced by the computation of weights based on current parameter values. The finite sample behaviour of the proposed method is investigated using both a Monte-Carlo experiment and a set of real data examples.

As it can be seen, the topics covered in this issue embrace a wide range of aspects of robust statistics. On Metron’s website it is listed: “The journal presents papers that approach statistical topics with originality; quality and clarity”. We thank all authors and all reviewers of this volume for their efforts to contribute to these goals. We hope that the readers of this special issue will consider that the papers it includes also provide an additional tribute to Professor Corrado Gini, the founder of Metron.

## Footnotes

1 Special report No 19/2017: Import procedures: shortcomings in the legal framework and an ineffective implementation impact the financial interests of the EU https://www.eca.europa.eu/Lists/ECADocuments/SR17_19/SR_CUSTOMS_EN.pdf
